# Sweet but sour: Impaired attention functioning in children with type 1 diabetes mellitus

**DOI:** 10.3389/fnhum.2022.895835

**Published:** 2022-09-08

**Authors:** Hayley M. Lancrei, Yonatan Yeshayahu, Ephraim S. Grossman, Itai Berger

**Affiliations:** ^1^Department of Pediatrics, Samson Assuta-Ashdod University Hospital, Ashdod, Israel; ^2^Faculty of Health Sciences, Ben-Gurion University of the Negev, Be’er Sheva, Israel; ^3^Pediatric Endocrinology Clinic, Samson Assuta-Ashdod University Hospital, Ashdod, Israel; ^4^Department of Education, Ariel University, Ariel, Israel; ^5^Pediatric Neurology Clinic, Samson Assuta-Ashdod University Hospital, Ashdod, Israel

**Keywords:** diabetes mellitus, type 1, ADHD, pediatrics, complications, brain development

## Abstract

Children diagnosed with type 1 diabetes mellitus (T1DM) are at risk for neurocognitive sequelae, including impaired attention functioning. The specific nature of the cognitive deficit varies; current literature underscores early age of diabetes diagnosis and increased disease duration as primary risk factors for this neurocognitive decline. Forty-three children with T1DM were evaluated for Attention Deficit/Hyperactivity Disorder (ADHD) symptomatology using the MOXO continuous performance test (MOXO-CPT) performed during a routine outpatient evaluation. The study cohort demonstrated a significant decline in all four domains of attention functioning. The effect was most pronounced with early age at T1DM diagnosis, a longer disease duration and with poorer glycemic control (represented by higher HbA1c values). With increased disease duration (of 5 plus years), acute hyperglycemia was associated with inattention in the real-time setting. These findings highlight the need for routine screening of neurocognitive function in children with T1DM so that early intervention can be employed during this crucial period of cognitive development.

## Introduction

Type 1 diabetes mellitus (T1DM) is one of the most prevalent chronic health conditions of childhood and adolescence with an increasing global incidence (Atkinson et al., 2014). The pathophysiology is based on the autoimmune destruction of pancreatic beta cells which creates a physiological deficiency of insulin (Zaccardi et al., 2016). This renders the patient reliant on exogenous insulin administration, an imperfect solution to achieving euglycemia. As a result, extreme fluctuations in blood glucose levels are common and with glucose acting as a primary fuel for the central nervous system, the potential impact on the growing brain cannot be overlooked.

Most published studies about cognitive performance among children with T1DM demonstrate an association between poor cognitive functioning and early onset of diabetes (particularly a diagnosis in children younger than 5 years of age) and/or a history of severe hypoglycemic episodes. The specific nature of the cognitive deficit varies, but verbal IQ, visuo-spatial/non-verbal functioning, memory, and attention have all shown to be affected (Hannonen et al., 2003; Gaudieri et al., 2008; Lin et al., 2010; He et al., 2018). Despite differing research methods, there appears to be an emerging consensus from the data, which is that children with T1DM are more likely to be at a cognitive and academic disadvantage. But specific recommendations regarding Attention Deficit/Hyperactivity Disorder (ADHD) among these children are absent.

In spite of this knowledge, current diabetes management guidelines do not advocate for any routine screening for cognitive impairment in pediatric patients (Kordonouri et al., 2014; Donaghue et al., 2018), nor for ADHD. Moreover, such neurocognitive complications are perpetuated as cognitive impairments including attention deficits further augment poor adherence and thus poorer glycemic control (Vinker-shuster et al., 2016).

Attention deficit symptomatology is characterized by significant inattention, sometimes with hyperactivity and impulsivity. In recent years, the apparent prevalence of this symptomatology has been increasing, underscoring the importance of this condition as a public health concern (Thomas et al., 2015). Affected individuals are at increased risk for lower academic achievement, social-peer difficulties, disruptive behavior, emotional and neuropsychological dysfunction (Berger et al., 2015). The cause is uncertain, being considered a multi-factorial disorder involving multiple causal processes and neuronal pathways (Berger et al., 2015). Two relevant hypotheses guided us in the preparation of this study:

1. There is a possible connection between T1DM and ADHD, as was suggested in a retrospective analysis of over 650,000 children and adolescents in German databases showing that ADHD was 40% more likely to be diagnosed among children with T1DM (Kapellen et al., 2016). A German multi-center registry study of over 56,000 children and adolescents found that those with both ADHD and T1DM suffered twice as often from diabetic ketoacidosis (i.e., poor metabolic control) compared with diabetic patients without ADHD (Hilgard et al., 2017).
2. Given that processing speed is a fundamental cognitive ability, we hypothesized that focusing on the processing speed parameter can serve as a marker which can be measured as a significant weakness in patients with ADHD and T1DM and can be easily evaluated when using computerized performance tests (Peled et al., 2020).

This study was designed to investigate the impact of both acute and long-term dysglycemia on different domains of attention functioning in a pedatric cohort with T1DM in comparison to the healthy pediatric population. The study also aimed to identify the impact of disease variables on attention functioning including age at diagnosis, disease duration, method of insulin administration and long-term glycemic control as indicated by HbA1c. Given the current literature, we expected that children with T1DM would have worse neurocognitive outcomes overall, with early age of onset and increased disease duration acting as risk factors for poorer outcomes. We also hypothesized that acute hyperglycemia would also have a negative impact on attention functioning in the real-time setting.

## Materials and methods

### Patient demographics

Study participants were recruited from the pediatric diabetes outpatient clinic at Assuta-Ashdod University Medical Center in Israel, between June-October 2020.

Children aged 6–18 years with a diagnosis of T1DM were recruited to the study. Minimum disease duration was 1-month.

Children suffering from active neurological disease including epilepsy; intellectual disability or children requiring special needs education; or any psychiatric illness including those requiring chronic use of medications were excluded from the study. In order to accurately reflect the general population, children with a formal diagnosis of ADHD were not excluded from the study yet were instructed to refrain from using stimulants or any other ADHD-related medications on the day of testing, which owing to their short half-life, provided a sufficient wash-out period prior to evaluation.

Informed consent was obtained from parents of all participants. The study protocol was approved by the hospital Institutional Review Board (Helsinki committee).

### Study design

Study participants underwent the following evaluation.

#### Attention deficit/hyperactivity disorder rating scale

Attention deficit/hyperactivity disorder symptoms were assessed using a validated ADHD Rating Scale (ADHD-RS) that was administered to the accompanying parent(s) at the time of the visit (DuPaul et al., 2016). This 18-item scale incorporates changes to the Diagnostic and Statistical Manual of Mental Disorders [DSM-5^®^] (American Psychiatric Association [APA], 2013). Previous validation studies provide support for the construct validity of this ADHD-RS since it’s factor structure is compatible with the manner in which the DSM–5 conceptualizes ADHD. Based on the ADHD-RS questionnaire, the child must have six out of nine responses of “often” or “very often” for inattentive items of the questionnaire or six out of nine responses of “often” or “very often” for hyperactive-impulsive items, or nine positive responses for the total questionnaire items to meet the DSM-5 criteria for ADHD.

#### Blood glucose testing

A finger prick test using a blood glucometer (FreeStyle Freedom Lite) was performed within a 15 min window preceding the MOXO test, which itself lasted a similar time-frame as mentioned below. HbA1c was measured simultaneously using the Cobas B 101 (Roche) point of care system.

#### MOXO continuous performance task

Participants also performed continuous performance task (CPT) assessment using MOXO-CPT (Neuro-Tech Solutions Ltd., Rehovot, Israel) (Cassuto et al., 2013). The MOXO-CPT (Neuro-Tech Solutions Limited, Rehovot, Israel) is a standardized computerized test designed to diagnose ADHD-related symptoms. The MOXO-CPT task requires the child to sustain attention over a continuous stream of stimuli and to respond to a prespecified target. Unlike most existing CPTs, the MOXO has improved ecological validity by exposing the participant to visual and auditory stimuli that mimic everyday distractors (Cassuto et al., 2013; Berger et al., 2017). The test’s validity and utility in distinguishing children and adolescents with ADHD from their typically developing peers were demonstrated in previous studies (Berger et al., 2017).

The test consisted of eight stages (levels). Each level consisted of 53 trials (33 target and 20 non-target stimuli) and lasted 114.15 s. The total duration of the test was 15.2 min. In each trial, a stimulus (target or non-target) was presented in the middle of the computer screen for durations of 0.5, 1, or 3 s and was followed by a “void” of the same duration. This method enabled us to distinguish accurate responses performed in “good timing” (quick and correct responses to the target performed during stimulus presentation) from accurate but slow responses (correct responses to the target performed after the stimulus presentation; during the void period). These two aspects of timing correspond to the two different deficiencies typical to ADHD; responding quickly and responding accurately (National Institute of Mental Health, 2012). The child was instructed to respond to the target stimulus as quickly as possible by pressing the space bar once and only once. The child was also instructed not to respond to any other stimuli but the target, and not to press any other key but the space bar. Both target and non-target stimuli were cartoon pictures free of letters or numbers. Also, the test included six different environmental distractors, each of them could appear as pure visual (e.g., three birds moving their wings), pure auditory (e.g., birds singing), or as a combination of visual and auditory stimuli (birds moving their wings and singing simultaneously). Each distractor was presented on the screen for a different duration ranging from 3.5 to 14.8 s, with a constant interval of 0.5 s between two distractors. For each child, four CPT indices were recorded: Attention (number of correct responses to target stimuli, including the rate of omission errors), Timing (correct responses to target stimuli conducted on accurate timing), Hyperactivity (a measure of motor activity) and Impulsiveness (responses to non-target stimuli, including the rate of commission errors).

Participants completed the MOXO-CPT in a standardized environment. The test was administered by a research co-ordinator. After a thorough explanation of the test procedure, patient understanding was verified *via* a short practice exam. Two versions of the test were in use, one for children and the other for adolescents and adults, with a test duration of 15 and 18-min respectively. For each domain, test scores were reported as standard deviations from the normal as compared to matched age and gender controls obtained in from previous studies (Berger and Cassuto, 2014). These standard deviations are translated into a “rank” from 1 to 4, with rank 4 indicating a pathological result, >-1.65 standard deviations from the mean. Obtaining “rank 4” in three or more domains of attention is considered an overall *positive* test, consistent with ADHD symptomatology (Cohen-Cymberknoh et al., 2018).

Children in whom possible ADHD was identified based on either the ADHD-RS or MOXO-CPT were referred to a pediatric neurologist for supplemental evaluation.

### Statistical analysis

MOXO-continuous performance task scores of the study cohort were compared to test scores of healthy children without T1DM obtained from previous studies. A One-sample *t*-test compared the averages obtained by the current sample with a value of 0 defining the performance level of matched age and gender healthy control children. Cohen’s d was used to calculate effect sizes. We conducted a non-parametric chi square for goodness of fit test to test the frequency of positive MOXO results in our cohort compared to the general population, according to the four attention domains. The general frequency of ADHD in the population is 5–7.2% (American Psychiatric Association [APA], 2013; Thomas et al., 2015). However, to be strict we used a point of comparison of 8%. Crosstabs chi square test was conducted in order to confirm convergence of cases receiving a positive overall result both on the ADHD questionnaire and the MOXO test. Pearson correlation was performed to examine the effect of several disease parameters including age of diagnosis, disease duration, diabetes control as indicated by HbA1c and method of insulin administration on attention functioning. Linear regression with 2 steps was used in order to control for current age. MOXO results were also analyzed with pre-test blood sugar levels to assess the real-time impact on attention functioning. A *p*-value of 5% or less was considered statistically significant. The analysis was performed in SPSS version 25.

## Results

From a total of 51 eligible study participants, 49 children were approached, of which 43 were successfully recruited completing the study task. This accounted for 84% of total eligible outpatient patients.

The study cohort included 23 males (53.5%) and 20 females (46.5%) (Table 1). Children were aged 6–18 years old (median age 11.95 years, SD 2.9). Mean body mass index (BMI) percentile was 58.6% (SD 28.9).

**TABLE 1:** Demographic characteristics of the study cohort (*n* = 43).

Gender (male), %	53.5
Age in years, mean (SD)	12.0 (±2.9)
Body mass index, centile, mean (SD)	58.6 (±28.9)
**Diabetes profile**	
Age at disease diagnosis in years, mean (SD)	8.1 (±3.7)
Duration of disease in years, mean (SD)	3.8 (±3.1)
Baseline HbA1c, %, mean (SD)	8.1 (±1.4)
Insulin administration *via* continuous subcutaneous infusion, %	72.1
History of hypoglycemic seizures, %	0
**Health profile**	
Diagnosed attention deficit disorder (according to a pediatric neurologist or psychiatrist), *n* (%)	3 (7.0)
Children receiving daily stimulant therapy, *n* (%)	2 (4.7)
Active neurological disease, %	0
Known IQ deficiency or requiring special needs education, %	0
Diagnosed psychiatric disorder, %	0

With regards to T1DM disease status, the average age at diagnosis was 8.1 years (SD 3.7) with a disease duration of 3.8 years (SD 3.1) at the time of recruitment. Average HbA1c was 8.1% (SD 1.4). Seventy-two percent of children received insulin *via* subcutaneous continuous insulin therapy. No study participants reported a history of hypoglycemic seizures.

### Results of attention deficit/hyperactivity disorder rating scale

Nine children (20%) were found to be *positive* on the ADHD-RS, a value more than two times the general population. Of these positive children, eight met the criteria for inattention (scoring more than 6 out of 9), whereas only four of the nine children met the criteria for hyperactivity-impulsivity.

### Results of the MOXO continuous performance task

Of the 43 children that completed the MOXO-CPT, nine had a *positive* test indicative of ADHD symptomatology, representing 20% of the total cohort.

Lower performance, as demonstrated by standard deviations from the norm, was seen in all four domains of attention functioning, particularly slowed timing (*p* = 0.000). A multivariate analysis of variance (MANOVA) with the four dependent variables as a joint DV demonstrated a significant effect [Wilks’ Lambda = 0.201, *F*_(4.39)_ = 38.86, *p* < 0.001, η^2^ = 0.80]. Subsequent one sample t-tests showed that scores in the timing domain were significantly different than the expected population, while other domains approached statistical significance (Table 2). The effect size was large for timing (d = 1.92) and small to moderate for attention (d = 0.26), impulsivity (d = 0.29) and hyperactivity (d = 0.30). When we considered the results as number of participants obtaining positive results, children had a higher frequency of *positive* tests (that is, obtaining “rank 4”) in all domains of attention (Table 3).

**TABLE 2:** MOXO continuous performance test (MOXO-CPT) scores of the overall study cohort, detailed in standard deviations from the healthy non-diabetic age and gender matched population.

	Mean MOXO score in standard deviations (standard deviation)	*P*-value for one sample *t*-test	95% CI	Effect size (Cohen’s d)
Attention	–1.62 (±6.25)	0.096	–3.55–0.30	0.26
Timing	–3.47 (±1.81)	0.000	–4.03-2.92	1.92
Impulsivity	–1.29 (±4.42)	0.062	–2.65–0.07	0.29
Hyperactivity	–0.81 (±2.69)	0.055	–1.64–0.02	0.30

**TABLE 3:** Rate of *positive* MOXO continuous performance test (MOXO-CPT) scores in the study cohort, defined as a score of “rank 4.”

	Number of diabetic children with positive results in domain	χ^2^ (*P*-value)
Attention	8	6.57 (0.010)
Timing	37	355.88 (0.000)
Impulsivity	12	23.15 (0.000)
Hyperactivity	12	23.15 (0.000)

Observed number of positive results in each domain was compared to an expected number of 3.44 which represents an upper limit of 8% in the general population.

#### Effect of type 1 diabetes mellitus disease variables on attention functioning

Increased T1DM disease duration was associated with poorer attention (*r* = –0.46, *p* = –0.002) and timing (*r* = –0.50, *p* = 0.001). After controlling for current age, disease duration continued to have a negative effect on attention (Beta = –0.47, *p* < 0.01) and timing (Beta = –0.42, *p* < 0.01).

Earlier age of T1DM diagnosis was correlated with reduced attention (*r* = 0.32, *p* = 0.038) as well as hyperactivity (*r* = 0.29, *p* = 0.056). There was no correlation between insulin administration method (injection vs. pump) on MOXO score (*p* = 0.683). Similarly, no gender differences were observed.

Increased HbA1c was associated with reduced attention (*r* = –0.49, *p* = 0.001) and poorer timing (*r* = –0.33, *p* = 0.031). This association stemmed from children with disease duration of 5 or more years (*n* = 14; *r* = –0.83, *p* = 0.001) compared to the children with disease duration less than 5 years (*n* = 29; *r* = −0.28). Impulsivity and hyperactivity were not impaired. Also, HbA1c was not associated with an overall *positive* MOXO test (*p* = 0.110).

### Convergence between attention deficit/hyperactivity disorder-rating scale and MOXO-continuous performance test

The two methods of ADHD testing, namely ADHD-RS and MOXO-CPT, tended to categorize the same cases as having or not having positive results. Twenty-nine children had negative results by both testing tools, another 4 were positive by both tools and 10 were positive on one and negative on the other [χ^2^_(1)_ = 3.803, *p* = 0.05].

We further compared the MOXO attention domains to the sub-categories of the questionnaire. Of the 43 study participants, 30 were identified as negative for inattentive symptomatology by both testing methods and 5 scored positive on both tools [χ^2^_(1)_ = 8.246, *p* < 0.01]. Regarding hyperactivity-impulsivity, 32 children were identified as negative and three scored positive on both tools [χ^2^_(1)_ = 5.219, *p* < 0.05].

### Real-time blood sugar level and MOXO results

Acute hyperglycemia (blood sugar level > 11.1 mmol/L) had no significant effect on MOXO test results in all four domains of attention.

A sub-analysis of children with T1DM duration of 5 or more years (*n* = 14) showed an inverse correlation between hyperglycemia (blood sugar level > 11.1 mmol/L) and attention functioning (Pearson correlation *r* = –0.53, *p* < 0.05). Pearson correlations for acute hyperglycemia with the other three domains of attention functioning yielded non-significant results.

## Discussion

Previous studies have examined the coexistence of a dual diagnosis of T1DM and impaired attention functioning (Desrocher and Rovet, 2004; Gaudieri et al., 2008; Naguib et al., 2009). However, the published data is still limited and there are no concrete published practice guidelines to facilitate high-quality care of patients with ADHD and T1DM, providing a rational, scientific approach to management that optimizes health outcomes and safety in this population. Our study cohort with T1DM demonstrated substantially higher rates of ADHD symptomatology. Twenty percent of participating children had a *positive* ADHD-RS questionnaire and 20% had a *positive* MOXO-CPT. This is more than double the prevalence of ADHD in the healthy pediatric population, ranging from 5 to 7.2% (American Psychiatric Association [APA], 2013; Thomas et al., 2015). As illustrated in Table 1, 7% of the study cohort had a previous diagnosis of attention deficit disorder according to a pediatric neurological or psychiatric evaluation. Thus in accordance with our study results, more than half of the study cohort with positive ADHD symptomatology was likely underdiagnosed. Our study results strengthen the primary hypothesis that ADHD is more likely to be diagnosed among children with T1DM, even among those with relatively shorter duration since T1DM diagnosis.

These results were statistically significant in all four domains of attention-test functioning (Table 3), with processing speed being most significantly impaired according to effect size. The findings were most pronounced with early onset disease, increasing disease duration and higher HbA1c levels.

There is a growing consensus of the negative impact of poor glycemic control on the immature and growing brain. Several hypotheses explaining this phenomenon have been proposed including the inhibition of myelin formation due to hyperglycemia as well as neuronal cell death due to excitotoxic and apoptoxic processes in the context of hypoglycemia (Fujoika et al., 1997; Malone et al., 2006, 2008). Although recurrent and chronic episodes of hypo- and hyper-glycemia respectively may lead to neuronal damage, the exact nature and magnitude of this damage is controversial.

Two large meta-analysis of children with T1DM support this neurocognitive compromise, demonstrating lower scores across most cognitive domains including attention and processing speed (Gaudieri et al., 2008; Naguib et al., 2009). One of the most influential risk factors for this impairment appears to be early age at diabetes diagnosis, in particular age 7 and below (Desrocher and Rovet, 2004; Gaudieri et al., 2008). Our study produced similar results, finding a statistically significant correlation between early age at diagnosis with inattention and hyperactivity.

Disease duration is another important variable associated with neurocognitive outcome; however, the precise onset of this decline is not straightforward. Subtle differences in cognition may already be observed from as early as 2 years of disease onset, particularly in the areas of intellectual ability and executive functioning (Cato et al., 2014). The neurocognitive gap rises with increasing disease duration. Rovet and Ehrlich (1999) followed a cohort of 16 children with T1DM over a 7-year period from their diagnosis. After 3 years of disease, a decline in verbal and visuospatial skills was reported. In a larger cohort study, children with T1DM statistically show poorer attention, processing speed, long-term memory and intelligence already 6-years post diabetes diagnosis (Northman et al., 2001).

Our study cohort, which dissimilar from existing literature included children with a relatively short disease duration (mean 3.8 years), showed a collective decline in all domains of attention, independent of age at MOXO testing, highlighting that the negative influence on neuropsychiatric functioning likely begins sooner in the disease course than previously anticipated. Our study also strengthened our hypothesis describing a unique effect that processing speed measurement might serve as a sensitive marker for cognitive ability among ADHD children.

While the cumulative effects of fluctuating blood sugar levels are more clear-cut, the singular effects have not been adequately examined. Acute hyperglycemia in T1DM children increases impulsivity (measured *via* a continuous performance task), yet other domains of attention remain largely unaffected (Rovet and Alvarez, 1997). In the adult population, cognitive function in T1DM patients is generally well-preserved during significant hyperglycemia (Draelos et al., 1995). Consistent with current literature, our study did not demonstrate an association between acute hyperglycemia and attention functioning. However, among study participants with increased disease duration (more than 5 years), acute hyperglycemia significantly impaired attention functioning in the real-time setting, suggesting an interplay between the acute and chronic effects of dysglycemia. Given that our study cohort primarily included children with short disease duration, it is possible that the acute effects of hyperglycemia on real-time attention were unable to be adequately assessed.

Although relatively small in sample size, the study cohort is generally reflective of the larger population. The high recruitment rate of almost 85% is a key factor in minimizing selection bias. The cohort is ethnically diverse, with participants affirming European and North African backgrounds. Mean glycosylated hemoglobin levels (HbA1c) are consistent with the current literature (Gerstl et al., 2008).

With regards to study limitations, information regarding socioeconomic status and family medical history of study candidates was not obtained. Second, although being a routine test for children with diabetes, we are unable to quantify the degree of associated anxiety from the finger prick test performed prior to testing and thus identify whether it influenced performance functioning in the MOXO-CPT. In saying this, as our study attempted to assess the day-to-day impact of diabetes and its management on attention functioning, finger prick testing prior to our assessment created an opportunity to mimic everyday life, increasing the relevance of our results to the study population. Third, due to the small sample size, regression for each disease variable was not suitable.

Of note, 72 percent of our cohort received insulin *via* continuous subcutaneous insulin infusions. This is greater than the international average, ranging from 39 to 45% in non-European and European countries respectively (Szypowska et al., 2016). However, we demonstrated that diabetes control (measured by HbA1c) was not influenced by the methods of insulin administration (*p* = 0.549) and therefore do not believe that this should impact on the study findings.

These findings are of large practical importance. ADHD symptomatology is likely to impact on routine everyday decisions that are crucial for diabetes management. Completion of tasks is likely to be influenced by inattention, whereas hasty decision making may result from hyperactivity and impulsivity (Cohen-Cymberknoh et al., 2018). Children with ADHD in addition to T1DM were shown to have higher overall HbA1c levels, higher withdrawal rates from insulin pump therapy, more presentations to emergency departments, longer hospitalizations and almost double the annual medical costs (Vinker-shuster et al., 2016). As time progresses, the neurocognitive sequalae worsen, further augmenting poor diabetes control, thereby creating a vicious cycle.

Pediatric patients tend to remain intentionally hyperglycemic so to prevent the potentially life-threatening complications of hypoglycemia, a theory known as “the fear of hypoglycemia” (Gonder-Frederick et al., 2011). This is particularly relevant in primary school children who are less independent in their diabetes management. During this crucial period of learning, children with T1DM of > 5 years duration are placed at an additional disadvantage, with acute hyperglycemia being associated with inattention.

Our study suggests that ADHD symptomatology may be substantially higher among the pediatric cohort diagnosed with T1DM compared to the healthy population. There are currently no recommendations for routine screening of cognitive impairment in children with T1DM. Yet it is clear that intervention is essential. We hope that the current study will trigger further investigations into preventative measures that will prevent the perpetuation of the already apparent health and educational gap among children with T1DM.

## Data availability statement

The original contributions presented in this study are included in the article/supplementary material, further inquiries can be directed to the corresponding author.

## Ethics statement

The studies involving human participants were reviewed and approved by The IRB of Assuta Ashdod Hospital. (Helsinki Committee). Written informed consent to participate in this study was provided by the participants’ legal guardian/next of kin.

## Author contributions

HL conducted the research project, collected the study data, reviewed the existing literature, and drafted the initial manuscript. YY conceptualized the study and reviewed the final manuscript. EG analyzed the study data. IB designed the study methods and reviewed the final manuscript. All authors have read and approved the final manuscript.
